# Timely initiation of complementary feeding and associated factors among mothers having children aged 6 to 24 months in North-West Ethiopia: a comparative cross-sectional study

**DOI:** 10.1186/s12887-024-04906-9

**Published:** 2024-07-03

**Authors:** Tilahun Kegne, Yihun Mulugeta Alemu, Gizachew Tadesse Wassie

**Affiliations:** Department of Epidemiology and Biostatistics, School of Public Health, College of Medicine and Health Sciences, Bahir Dar University, Bahir Dar, Ethiopia

**Keywords:** Child nutrition, Complementary feeding, Demographic disparity, Ethiopia, Farta District

## Abstract

**Background:**

The timely introduction of complementary foods during infancy is necessary for nutritional reasons, and to enable the transition from milk feeding to family foods. In the past years, despite efforts that have been put to increase the utilization of timely initiation of complementary feeding practice in Ethiopia, improvements are not satisfactory.

**Objective:**

To compare the prevalence of timely initiation of complementary feeding and its associated factors among mothers who have Children 6–24 months in Debre Tabor town and rural Farta district, North-west Ethiopia, 2021.

**Methods:**

A community-based comparative cross-sectional study was employed from December 1/2020 to 30/ 2020 among 1100 mothers. Data were collected using a structured questioner and analyzed using Statistical Product and Service Solutions. Logistic regression analysis with a 95% confidence interval carried out to determine the association between explanatory and the outcome variables. A P-value of < 0.05 was considered statistically significant.

**Results:**

The prevalence of timely initiation of complementary feeding among urban and rural mothers was 69.8% with (95% CI: 66%, 74%) and 51.9% with (95% CI: 48- 56%) respectively. Urban residence [AOR = 1.39, 95% CI: (1.02–1.94)], had antenatal care visits [AOR = 0.24 (95%CI: (0.13, 0.44)], had post natal care checkups [AOR = 0.44, 95%CI: (0. 27- 0.72)] and being a governmental employee [AOR = 2.82; 95% CI: (1.91–6.1)] were factors associated with timely initiation of complementary feeding among urban mothers. Whereas in rural settings: institutional delivery [(AOR = 2.21, CI: 1.35–3.65)], post natal care checkups [(AOR = 0.53, CI: (0.36–0.77)] being daily laborer [AOR = 3.47; 95% CI: (1.78–6.75)] were associated with timely initiation of complementary feeding.

**Conclusion:**

The prevalence of timely introduction of complementary feeding in children aged 6–24 months is still low in the study areas. There was also disparity between urban and rural mothers in which urban mothers practiced better.

## Background

The timely initiation of additional foods during infancy and early childhood is necessary for a healthy developmental process [1]. At the age of 6 months, infants need for energy, and micronutrients start exceeding what breast milk provides. They are developmentally ready to start complementary food [2]. Complementary foods (CF) are defined as any solid or liquid foods with a nutritional value other than breast milk, offered to breastfed infants [3].

Exclusive breastfeeding (EBF) for 6 months should be complemented by offering solid foods besides breast milk. This has been recommended since 2002 by the World Health Organization (WHO) for optimum infant feeding practice [4]. It is a significant milestone that has nutritional, developmental, and health implications [5, 6].

Physical, cognitive, and social development occur rapidly within the first two years of life, requiring proper nutrition [7]. Besides, many physiological processes have an effect on early growth and development [8, 9]. It is also well known that it is a “critical window” for the promotion of healthy growth and behavioral development [10]. The WHO also promotes and supports the implementation of the Infant and Young Child Feeding (IYCF) strategy as a critical element of efforts to address child malnutrition and mortality [11].

There is considerable evidence that various inappropriate complementary feeding practices, including the untimely introduction of complementary foods, irregular feeding frequency, and low dietary diversity of complementary foods, negatively impact children’s health [12–16].

The Ethiopian Ministry of Health has been implementing the WHO recommendation that infants and children be exclusively breastfed for the first 6 months of life with no additional liquids or foods given [17, 18]. The major indicators of under nutrition among under-five children in Ethiopia were stunting (40%), wasting (11%), underweight (23%), and micronutrient deficiency [19–21].

More than 800,000 deaths in children could be prevented by scaling up the optimal timing of CF practices. A key outcome of an appropriate and timely initiation of CF is a reduced risk of future-onset communicable diseases [22]. Despite international guidelines and programs to improve IYCF practices, < 40% of infants are exclusively breastfed worldwide, and only approximately 60% of children aged 6–8 months receive appropriate and safe complementary foods, reflecting deficiencies in global IYCF, Ethiopia has shown important evolution in dropping under-five, infant, and neonatal mortality rates by 47%, 39%, and 25%, respectively [23].

In spite of its achievements, Ethiopia has still encountered children’s infections and malnutrition. This is believed to be mainly due to inappropriate child feeding practice and food insecurity. There have been studies done in Ethiopia regarding this research problem [24, 25], but none of them have been able to identify the urban rural disparities of prevalence and contributing factors [26]. To understand the influence of various levels of societal environments such as individuals, (mothers), family, community, cultural norms, and health care related factors on mothers’ behaviors related to timely initiation of complementary feeding, ecological systems theory was applied. This theory looks at a child’s development within the context of the system of relationships that form his or her environment. Therefore, this study aimed to compare the prevalence of timely initiation of CF and its associated factors among mothers of children aged 6 to 24 months children and to inform the development of effective interventions to improve complementary feeding practices in North-west Ethiopia.

## Methods and materials

### Study area and period

The study was conducted in two selected areas, Debre Tabor (urban) and Farta district (rural) residents, from December 1st, 2020 to December 30th 2020. Debre Tabor town is the capital city of the South Gondar zone which is one of the 11 zones, found in the Amhara Regional State. As to south Gondar human resources, the town have an estimated total population of 67, 004 and the district has 250,731 inhabitants. Of these, 34, 246 and 54, 406 of them were women, respectively for urban and rural.

### Study design

A community-based comparative cross-sectional study was conducted.

#### Inclusion criteria of study participants

All mothers with children aged from six months to two years in the Debre Tabor urban and Farta district rural area were included.

### Study variables

#### Dependent variable

Timely initiation of complementary feeding.

#### Independent variables

Socio-demographic characteristics of mothers: age, sex, residence, occupational status, educational status, marital status, religion, and income.

Maternal care and birth history: antenatal care (ANC) checkup status and number of visits; postnatal care (PNC) checkups; place of delivery; and number of live birth; birth interval, and multiple pregnancies.

Utilization of the media, information about infant feeding and media exposure were factors tested.

#### Operational definitions

Complementary feeding: provision of solid or liquid foods along with breastfeeding in the period between 6 months to 2 years [27, 28].

Timely initiation of complementary feeding: It is the initiation of additional supplementary foods which are rich in energy, protein, and micronutrients (particularly iron, zinc, calcium, vitamin A, vitamin C, and folate), not spicy or salty, staple cereals, roots, and starchy fruits for young children at six months of age, along with continued breastfeeding [29].

#### Sample size determination

The sample size was calculated based on a double population proportion formula by using Epi Info version 7.2.01s and considering the following assumptions: 80% power, 95% confidence level for factors associated with timely initiation of complementary feeding taken from previous works of literature. The maximum sample size was obtained from mothers’ educational status [30] which gives 334, then multiplied by a design effect of 1.5 and adding 10% the non-response rate given 1100 study participants. Therefore, the final total sample size was 1100 (550 urban and 550 rural) mothers who had six to 24 month- old were included in the study.

### Sampling procedures

A multi-stage stratified sampling technique was employed to select the study participants. First, three out of six urban and eight out of 36 rural kebeles in Debretabor and Farta districts were selected randomly (lottery method) respectively. Numbers of participants were proportionally allocated to each selected kebele based on the number of households. In the second stage, a systematic random sampling technique was employed to selected mothers at each selected kebeles. The sampling frame was obtained from the health extension workers’ logbooks of the respective kebeles. Finally, an equal number of study participants were selected by using systematic random sampling (every 3rd interval for urban and 4th interval for rural and the random start was determined by lottery method) from each selected kebeles and 1100 households were selected from both urban and rural residents.

### Data collection tools

Standardized, structured interviewer-administered questionnaires were used to collect data. The questionnaires were adopted by reviewing different literatures, which is composed of socio-demographic and economic characteristics, health care, and complementary feeding practice-related variables. Complementary feeding related factors were assessed according to the key indicators recommended by the WHO and the IYCF strategy of Ethiopia.

### Data management and analysis

Data were cleaned (checked for errors, impossible, unlikely, and not consistent values, edited, and post coded for computerization) and entered into Epi Data Version 4.2.01 statistical software and exported to SPSS Version 23 statistical software for analysis. Data were presented using text, frequencies, and tables. A Binary logistic regression model was used to identify the factors associated with a timely initiation to complementary feeding. Separate models were run for the overall population, urban and rural residents. Variables with p-values of < 0.2 in the bivariable analysis were entered into the multivariable logistic regression analysis. Adjusted odds ratios (AOR) with their 95% CI were reported, and statistical significance was determined at *p* < 0.05. Multicollinearity was checked using variance inflation factor (VIF) with maximum threshold 10 and no multicollinearity was detected.

### Data quality assurance

Data quality was ensured by providing one day of training for data collectors and supervisors about the overall data collection techniques. Two BSc nurses were recruited as supervisors, and four diploma nurses were recruited as data collectors. The questionnaire was first prepared in the English language, then translated to the local language, Amharic, and retranslated back to English. A Pre-test was conducted outside of the study area in a similar setting. Onsite-checking and reviewing of the completed questionnaire were done by the principal investigator and supervisors to ensure the completeness and clarity of the information.

## Results

### Socio-demographic and economic characteristics study participants

A total of 1072 (536 from each setting) mothers with children aged 6 to 24 months took part in the survey, with a similar response rate of 97%. The majority of the mothers were Orthodox Christians 302 (56.3%) in urban and 408 (76.1%) in rural. Two hundred two (37.7%) urban, and forty seven (8.8%) of rural respondents had college and above educational status (Table 1).

**Table 1:** Socio-demographic characteristics among mothers who had 6–24 months children in North-West Ethiopia, 2020 (*N* = 1072)

Variables	Categories	Urban((*n* = 536)		Rural (*n* = 536)	
		Frequency (Un weighted)	%	Frequency (Un weighted)	%
Age of the mother in years	15–19	14	2.6	36	6.7
	20–25	138	25.7	157	29.3
	26–29	145	27.1	90	16.8
	>=30	239	44.6	253	47.2
Marital status	Single	91	17	36	6.7
	Married	349	65.1	343	64.0
	Divorced	41	7.6	43	8.0
	Widowed	38	7.1	47	8.8
	Separated	17	3.2	67	12.5
Religion	Orthodox	302	56.3	408	76.1
	Muslim	107	20.0	84	15.7
	Protestant	97	18.1	31	5.8
	Other	30	5.6	13	2.4
Educational status of mother	Unable to read and write	37	6.9	115	21.5
	Able to read and write	47	8.8	266	49.6
	Primary	141	26.3	86	16.0
	Secondary	109	20.3	22	4.1
	Diploma and above	202	37.7	47	8.8
Educational status of spouse	Unable to read and write	24	4.5	131	24.4
	Able to read and write	66	12.3	217	40.5
	Primary	76	14.2	144	26.9
	Secondary	136	25.4	21	3.9
	Diploma and above	234	43.7	23	4.3
Occupation of mother	Housewife	36	6.7	275	51.3
	Self employed	74	13.8	121	22.6
	Daily labor	37	6.9	83	15.5
	Merchant	73	13.6	15	2.8
	Governmental employee	307	57.3	31	5.8
	Other	9	1.7	11	2.1
Occupation of spouse	Farmer	32	6.0	405	75.6
	daily laborer	84	15.7	58	10.8
	Merchant	206	38.4	53	9.9
	Governmental employee	207	38.6	7	1.3
	Other	7	1.3	13	2.4
Access to radio	Yes	389	72.6	229	42.7
	No	147	27.4	307	57.3
Television	Yes	281	52.4	41	7.6
	No	255	47.6	495	92.4
Read magazines and books (at least one month)	Yes	329	61.4	41	7.6
	No	207	38.6	495	92.4
Monthly income of the household	<= 500 birr	88	16.4	192	35.8
	501–1000 birr	113	21.1	170	31.7
	1001–1500 birr	68	12.7	4	0.7
	1501–2000 birr	29	5.4	87	16.2
	>= 2001 birr	238	44.4	83	15.5
Number of family members	2–3	510	95.1	404	75.4
	4–6	26	4.9	132	24.6
	> 6	8	1.5	29	5.4
Sex of the child	Male	328	61.2	212	39.6
	Female	208	38.8	324	60.4
Age of the child in month	6–10 months	138	25.7	96	17.9
	11–15 months	266	49.6	256	47.8
	16–20 months	87	16.2	98	18.3
	21–24 months	45	8.4	86	16.0

### Maternal reproductive history and health service utilization

Regarding reproductive history, 465 (86.8%) and 432 (80.6%) of the mothers were primiparous in urban and rural areas respectively. Most of urban mothers 463 (86.5%) and 313 (58.7%) of rural mothers had antenatal care visits. Nearly half of 301 (56.2%) urban and two –third 376 (70.1%) of rural mothers gave birth at governmental health facilities (Table 2).

**Table 2:** Maternal reproductive history and health service utilization in North-West Ethiopia, 2020 (*N* = 1072)

Variables	Categories	Districts			
		Urban (*n* = 636)		Rural (*n* = 636)	
		Frequency (Un weighted)	%	Frequency (Un weighted)	%
Number of live births	<=2	430	80.2	315	58.8
	3–4	94	17.5	185	34.5
	>=5	12	2.2	36	6.7
Birth interval	<= 2 years	142	26.5	273	50.9
	>= 3 years	394	73.5	263	49.1
ANC visits	Yes	463	86.5	313	58.4
	No	73	13.5	223	41.6
Number of ANC visit	1–2 times	236	72	194	35.6
	>=3 times	244	48	185	34
Told about time of initiation of CF during ANC visit	Yes	314	81	265	68
	No	74	19	123	32
Place of current delivery	Home	104	19.4	92	17.2
	Government HF	301	56.2	376	70.1
	private HF	97	18.1	47	8.8
	Other	34	6.3	21	3.9
PNC checkups	Yes	424	79.1	262	48.9
	No	112	20.9	274	51.1
Parity	Primiparous	465	86.8	432	80.6
	Multiparous	71	13.2	104	19.4

*Note* ANC; Antenatal care, PNC; Post natal care, CF; Complimentary feeding, HF; Health facility

### Timely initiation of complementary feeding in the districts

The overall prevalence of timely initiation of CF was 60.8% [95% CI: ( 57.9–63.8)].Whereas, it was 69.8%, 51.9% in urban and in rural settings respectively (Table 3).

**Table 3:** Maternal and child health, and source of information of mothers about timing of introducing complementary foods to their children in in North-West Ethiopia (*N* = 1072)

Variables	Categories	Districts			
		Urban(*n* = 636)		Rural (*n* = 636)	
		Frequency (Un weighted)	%	Frequency (Un weighted)	%
Counseling on CF	Yes	421	78.5	317	59.1
	No	115	21.5	219	40.9
Source of information about initiation of CF at six months of age	Colleagues	55	10.3	62	11.6
	Family	63	11.8	64	11.9
	Health extension workers	193	36.0	202	37.7
	Governmental health facility	75	14.0	63	11.8
	Private health facility	88	16.4	80	14.9
	Media	53	9.9	52	9.7
	Others	9	1.7	13	2.4
Give the child pre-lactation food/fluid	Yes	312	58.2	318	59.3
	No	224	41.8	218	40.7
Kinds of commonly introduce pre-lactation foods	Butter	138	25.7	129	24.1
	Sugar solution	64	11.9	70	13.1
	Cow’s milk	75	14.0	78	14.6
	Water	15	2.8	14	2.6
	Other	20	3.7	27	5.0
Reasons for introducing pre-lacteal feed	Breast milk insufficient	104	19.4	139	25.9
	Culture of community	92	17.2	100	18.7
	Maternal illness	45	8.4	22	4.1
	Caesarean delivery	16	3.0	13	2.4
	Painful breast	37	6.9	21	3.9
	Others	17	3.2	24	4.5
Have you started CF at the age of 6 months	Yes	374	69.8	274	51.9
	No	162	30.2	262	49.1

### Factors associated with timely initiation of complementary feeding in the district

Bivariate analysis was performed to identify candidate variables for the multi variable logistic regression analyses at *P* < 0.2. Thus, mothers’ educational status and occupation, husbands’ educational status and occupation, residence, ANC checkups, PNC checkups and place of birth were candidate variables for the final model. From multivariate logistic regression analyses, maternal education, residence, ANC checkups, place of delivery, and PNC checkups were variables significantly associated with timely initiation of CF at a p-value of 0.05, with 95% CI.

Mothers with a diploma or higher educational status were 2.72 times more likely to start CF to their infants timely than those who couldn’t read and write [AOR = 2.72; 95% CI: (1.69–4.37)]. Mothers from urban areas were 1.39 times more likely to initiate CF timely than mothers from rural areas [AOR = 1.39, 95% CI: (1.01, 1.94)].

Maternal health care related factors were significantly associated with the timely initiation of CF in the district. Mothers who had no ANC visits were 41% less likely to initiate CF timely than those who had [AOR = 0.59 (95% CI = (0.43–0.81)]. Besides, mothers who gave birth at governmental health facilities were 2.37 times more likely to initiate CF timely [AOR = 2.37; 95% CI: (1.68–3.50)] as compared to those who gave birth at home. Moreover, those mothers who had postnatal care checkups were about 1.65 times more likely to timely initiate CF compared to their counterparts [AOR = 1.65; 95% CI: (1.24–2.21)] (Table 4).

**Table 4:** Factors association with timely initiation of complementary feeding in North-West Ethiopia, 2020(*N* = 1072)

Variables	Categories	Timely initiation of CF		COR(95% C.I)	AOR (95% CI)	P-value
		**Yes**	**No**			
Educational status of the mother	Unable to read &write	63	89	1	1	
	able to read & write	141	172	1.15(0.78–1.71)	1.67 (1.20-32.49)	0.012
	Primary	108	119	1.28(0.84 − 0.19)*	1.81(1.16–2.83)	0.009
	Secondary	65	66	1.39(0.86 − 0.22)*	1.86(1.09–3.10)	0.021
	Diploma and above	150	99	2.14(1.42–3.22)*	2.72(1.69–4.37)	< 0.001
ANC visits	Yes	419	397	1	1	
	No	108	148	0.69(1.08–1.92)*	0.59 (0.43–0.81 )	< 0.001
Residence	Urban	374	162	2.14(1.66–2.75)*	1.39(1.01,1.94)	0.048
	Rural	278	258	1	1	
Place of delivery	Home	93	103	1	1	
	Gov’t HF	348	329	2.39(1.73–3.29)	2.37(1.68–3.5)	< 0.001
	private HF	71	73	1.60(1.04–2.46)	1.33(0.0.48–2.1)	0.217
	Other	15	40	1.76(0.95–3.24)	1.58(0.38–3.02)	0.158
PNC checkups	Yes	466	186	2.44(1.89–3.15)	1.65(1.24–2.21)	< 0.001
	No	207	213	1	1	

*; p- value < 0.2, for COR (95% C.I), ANC; Antenatal care, HF; Health facility, CF; Complimentary feeding, PNC; Post natal care

### Factors associated with timely initiation of complementary feeding among urban mothers

Mothers’ occupation, place of delivery, ANC visit, and PNC checkups were variables significantly associated with the timely initiation of complementary feeding in urban settings. Mothers who were government employee were 2.82 times more likely to initiate CF timely than housewives [AOR = 2.82; 95% CI: (1.91–6.09)], and who did not have ANC visits were 76% less likely to start complementary feeding timely than those who did have [AOR = 0.24 = 95% CI: (0.13–0.44)]. Similarly, those mothers who did not receive PNC checkups were 55% less likely to initiate CF timely than those who received the service (AOR = 0. 44, CI; 0. 28- 0.72) (Table 5).

**Table 5:** Factors associated with timely initiation of complementary feeding among mothers who had 6–24 months children in Debre Tabor Town, North-West Ethiopia 2020 (*n* = 536)

Variables	Categories	Timely initiation of CF		COR(95% C.I)	AOR (95% C.I)	P- Value
		**Yes**	**No**			
Mothers’ occupation	Housewife	62	213	1	1	
	Self-employed	102	19	2.09(1.058–4.15)*	3.24(1.51–6.94)	0.002
	Daily labor	43	40	4.83(1.80-12.93)*	8.32(2.83–8.46)	< 0.001
	Merchant	8	7	2.38(1.211–4.68)*	3.34(1.57–7.12)	0.002
	Government employee	14	17	2.82(1.681–4.73)*	3.40(1.91–6.08)	< 0.001
	Other	7	4	3.0(0.57-15.78)	1.65(0.23–8.64)	0.090
ANC	Yes	123	39	1	1	
	No	340	34	3.17(1.91–5.24)*	0.24(0.13–0.44)	< 0.001
PNC	Yes	112	50	1	1.	
	No	312	62	2.25(0.24–0.56)*	0.44(0.27–0.72)	< 0.001

*p- value < 0.2, for COR (95% C.I)

### Factors associated with timely initiation of complementary feeding among rural mothers

The mother’s occupation, spouse’s occupation, place of delivery, and PNC checkups were factors significantly associated with timely initiation of CF among rural residents. Mothers who were self-employed were 2.25 times more likely to initiate CF timely for their children than those who were house wife [AOR = 2.25; 95% CI: (1.35–3.76)]. Mothers whose husband occupation were daily laborers were 3.47 times [AOR = 3.47; 95% CI: ( 01.78–6.74)] and merchants were 4.78 times [AOR = 4.78 (95% CI: 2.04–11.19)] more likely to initiate CF timely than whose husbands were farmers. Similarly, mothers who gave birth in health facilities were 2.25 times more likely to initiate CF timely than those who gave birth at home [AOR = 2.25, 95% CI: (1.37–3.65)]. On the other hand those mothers who had no postnatal care utilization were 47% [AOR = 0.53 (95% CI: 0.36–0.77)] less likely to initiate CF timely than who had post natal care service (Table 6).

**Table 6:** Factors associated with timely initiation of complementary feeding among mothers who had 6–24 months children in rural Farta district, North-West Ethiopia, 2020 (*n* = 536)

Variables	Categories	Timely initiation of CF			COR(95% C.I)	AOR (95% C.I)	P value
		Yes	No				
Mothers’ occupation	Housewife	121		154	1	1	
	Self-employed	87		34	3.25(2.05–5.17)*	2.25(1.35–3.76)	0.002
	Daily labor	38		45	1.07(0.656–1.76)	0.79(0.46–1.36)	0.402
	Merchant	8		7	1.45(0.51–4.12)	1.02(0.32–3.28)	0.965
	Government Employee	14		17	1.04(0.49–2.21)	0.97(0.42–2.22)	0.955
	Other	10		1	9.72(1.60–5.79)	5.61(0.67–46.83)	0.111
Spouses’ occupation	Farmer	153		297	1	1	
	Daily laborer	86		56	3.21(1.75–5.89)*	3.47(1.78–6.74)	< 0.001
	Merchant	166		93	6.88(3.16–8.95)	4.78(2.04–11.19)	< 0.001
	Government Employee	122		99	0.92(0.20–4.15)	1.01(0.19–5.16)	0.988
Place of delivery	Home	36		62	1	1	
	Government health facilities	215		155	2.38(1.50–3.70)*	2.21(1.35–3.65)	0.002
	Private health facilities	19		28	1.16(0.573–2.38)	0.97(0.44–3.62)	0.080
	Other	8		13	1.06(0.40–2.80)	0.98(0.33–2.91)	0.650
PNC	Yes	104		154	1	1	
	No	158		120	1.95(0.36–0.72)*	0.53(0.36-0.77)	< 0.001

*p- value < 0.2, for COR (95% C.I)

## Discussions

This study revealed that the overall prevalence of timely initiation of CF was 60.8% [95% CI: ( 57.9, 63.8)]. It is much greater than previous findings from Iran (44.8%) [1], Nigeria (53%) [2], Bangladesh (7o%) [3], Kamba woreda (40.6%) [4], Halaba Kulito (57.8%) [5], and Damot Weydie District in Ethiopia (50.6%) [6]. But it was lower than studies from Dessie (65.1%) [7], and Addis Ababa, Ethiopia (83%) [8]. Meanwhile, this finding was in line with studies done in Hiwot Fana Specialized Hospital (60.5%), Mekelle (62.8%), Lalibela (63%) and Pawie, Ethiopia (61.8%) [9–12].

Complementary feeding was initiated on time among 69.8% of urban residents and 51.9% of rural residents. The reason could be that mothers from urban areas may have better access to health care and health information [17, 18]. The urban areas prevalence was in line with the studies done in Bangladesh (70%) [3], Addis Abeba (68%) [8], Sodo town (71.2%) [19] and Dessie Referral Hospital in Ethiopia (66.5%) [7]. But it was higher than studies conducted in Nigeria (53%) [2], Addis Abeba (55.2%) [14], and Mekelle (62.8%) [11]. The higher prevalence of timely initiation of CF in the current study areas could be related to the improvements in utilization of ANC and institutional delivery services. Hence, nutrition education and counseling are components of maternal health care services, so a better utilization of these services will bring an added benefit to improving mothers’ awareness of appropriate child feeding practices [20]. Unlike the urban areas, the prevalence of timely initiation of CF in rural areas (51.8%) was found to be lower than in coastal south India (63%) [21], Hiwot Fana specialized hospital (60.5%) [12], Pawie (60%) [22], Halaba Kulito (58%) [5], Demo Weydie (57%) [6] and Lalibela districts of Ethiopia (63%) [10]. The difference might be due to low socio-economic status, and low health care accessibility in rural areas.

Mothers’ education, residence, place of delivery, ANC visits, and PNC checkups, were the factors associated with the timely initiation of complementary feeding in the district. Mothers who had ANC visits, were delivered at health institution, and had postnatal care checkups were more likely to initiate CF timely than their counterparts. This finding was consistent with studies done in Hyderabad, Pakistan, Benin, Axum, Mekelle, Soro district, and Addis Ababa, Ethiopia [9, 11, 13–16]. This might be because during utilizing maternal health services, child nutrition counseling would be provided to the mothers. On the contrary, mothers who did not have ANC and PNC visits and were delivered at home would not have sufficient information about recommended child feeding practices. The study also revealed that mothers from urban areas initiated CF timely than mothers who live rural areas. This finding is in line with studies in Addis Abeba, Axum town, and Lalibela, Ethiopia [10, 13, 14].

In the separate model; occupation of mother, ANC and PNC visits were factors significantly associated with timely initiation of complementary feeding from urban setting; whereas occupation of mother, occupation of the spouse, place of delivery and PNC visits were factors associated with timely initiation of complementary feeding from rural setting.

In urban areas, self-employed, daily laborers, merchants, and government employed mothers were more likely to initiate CF timely than housewife mothers. This result was consistent with studies conducted in Benin, Mekele, and Lalibela, Ethiopia [10, 11, 15]. This might be because government employee mothers are usually educated and have access to information about the benefits of timely initiating CF for their children. The study also revealed spouses’ occupations had an association with the timely initiation of CF among rural mothers. Those mothers whose spouses occupation was daily laborers and merchants were more likely to initiate CF timely than those who are farmers. This finding was consistent with studies in other places: Pokhara, India, Ireland, Malawi, Gode district and Axum, Ethiopia [13, 23–27]. Mothers from urban areas who did not have ANC visit were less likely to initiate CF timely than those who had ANC visits during their pregnancy. This finding was in agreement with other reports in Ethiopia [9, 12–14, 28]. Obviously, pregnancy has been considered an important window of opportunity to deliver nutrition counseling and education to children [29]. The other predictor of timely initiation of complementary feeding was history of PNC checkups. In this regard, mothers who had a history of more than PNC checkups started complementary feeding more timely than that had not. This was in line with studies done in Pakista, Mekelle town, Gondar, and Lalibela Districts of Ethiopia [9, 10, 16, 30]. Visiting health institution creates greater opportunity to get health education related to complementary feeding type, benefit and appropriate timing of complementary foods initiation.

Mothers from rural areas who delivered at health institutions were more likely to initiate CF timely than those who delivered at home. The finding was supported by reports from India, Pakistan, Benin, Soro district, and Lalibela District, Ethiopia [9, 10, 16, 23]. Mothers who give birth at health institutions are believed to have better opportunity to access appropriate child feeding information, which probably improves their capacity to challenge unfavorable attitudes of the community [31, 32].

PNC utilized mothers from rural areas were also more likely to initiate CF timely than those who did not. Similar findings were reported from Benin, Axum Kamba district, and Dabat district, Ethiopia [4, 15, 33]. Different literatures identified postnatal period as an ideal time to counsel mothers on optimal complementary feeding practice [11, 34, 35].

The occupational status of the mother was one of the determinants of the timely initiation of complementary feeding practice among rural residents. Mothers who were self-employed were more likely to initiate timely complementary feeding than those who were housewives. This was consistent with studies conducted in Malawi, Kamba, and Lalibela, Ethiopia [4, 10, 24]. The study also revealed that mothers who had merchant spouses initiate CF more timely than those whose husbands were farmers. This finding was consistent with studies conducted in Hyderabad, Pakistan, Egypt, Gode district and Dabat District, Ethiopia [16, 33, 36, 37].

Strengths and limitations of the study.

The strength of the current study lay in its use of a large sample size, which has the potential to increase the study’s power. However, this study has limitations. Since women were asked retrospectively for their exposures, the response might be susceptible for recall bias. Furthermore, cross-sectional nature of the study does not allow for determination of causation between the independent variables and timely initiation of complementary feeding.

## Conclusions

The prevalence of timely introduction of complementary feeding in children aged 6–24 months is still low in the study areas. There was also a disparity between urban and rural mothers who practiced it. Mothers from urban areas were better practiced than mothers from rural areas. Maternal health service related factors; antenatal visit status, institutional delivery, and postnatal care checkups were associated with the timely initiation of complimentary feeding. Besides, mothers’ educational status, mothers’ occupation status and spouses’ occupational status were significantly associated factors. Hence, promoting the existing maternal health care services by giving special consideration to rural mothers would be helpful strategies for promoting the timely initiation of complimentary feeding practices.

## Data Availability

The datasets used and/or analyzed during the current study are available from the corresponding author on reasonable request.
